# An Assessment of Germicidal Ultraviolet Treatment Cabinets and Carousels Using a Bacteriophage Surface Challenge

**DOI:** 10.1089/apb.2023.0008

**Published:** 2023-12-05

**Authors:** Jodi Brookes, Alan Beswick, Iwona Rosa, Claire Bailey, Charlotte Beynon, Stephen Stagg, Neil Bennett

**Affiliations:** ^1^ Health and Safety Executive Science and Research Centre, Buxton, United Kingdom.; ^2^ The Animal & Plant Health Agency, Addlestone, Surrey, United Kingdom.

**Keywords:** UVC, carousel, cabinet, Phi6, PAPR, irradiation

## Abstract

**Introduction:**

Modern germicidal ultraviolet C (UVC) equipment can deliver automated UV disinfection treatment by predetermined or self-monitoring cycle. Limited information exists about the performance of such UV systems for treating SARS-CoV-2 and other viral contaminants on surfaces. Published studies differ in their approaches due to the absence of an approved test method.

**Methods:**

The ability of germicidal UVC irradiation systems to disinfect surfaces at room and cabinet scale was assessed. Test carriers, seeded with bacteriophage Phi6, were irradiated following a new standard test method. Powered air-purifying respirator equipment was then used to introduce a more demanding challenge.

**Results:**

Treatments of seeded carriers using UVC cabinets gave Phi6 log reductions up to 4.58 logs, with little difference between systems. Subsequent treatments, with carriers located on respirator ensembles, were similar, despite shadowing effects. Differences existed for various combinations of cabinet and carrier location. The Phi6 log reduction range was slightly wider for carousel systems, with the most exposed carrier positions giving the greatest Phi6 reductions for seeded respirators.

**Discussion:**

Cabinets demonstrated similar performance despite different technical specifications, with maximum observed Phi6 reduction indicating a measurable level of efficacy. There was a more obvious difference in performance between the two carousels, where one delivered an almost twofold higher UVC dose than the other, the most likely explanation for observed performance differences.

**Conclusion:**

UVC cabinets and carousels demonstrated Phi6 reductions that could augment routine cleaning measures for reusable respirators. In real-world scenarios, germicidal UVC devices could therefore potentially offer benefits for reducing contact transmission from infectious viruses.

## Introduction

Ultraviolet (UV) radiation is emitted at wavelengths of between 100 and 400 nm. The lower threshold for ultraviolet C (UVC) lies at 100 nm, with the upper end of the range at 280 nm. However, difficulties associated with UVC transmission below 200 nm means that the lower practical limit for UVC applications in air is 200 nm. Within this accepted UVC range, the most commonly studied germicidal UVC applications are those emitting at 254 nm.^1–3^

The design of UVC disinfection equipment depends on application and includes those used for water treatment, whole room surface treatment, small object surface treatment, ceiling mounted air treatment, and systems integrated within ventilation ducts for the treatment of airborne contaminants.^3–8^

The germicidal treatment of water and air is outside of the scope of this study, and of primary interest was an evaluation of UVC germicidal performance of surface treatments using floor standing carousels and worktop cabinets. These system types both work by delivering a controlled UV treatment to achieve surface disinfection by a pre-set or self-monitoring cycle.^7,9^

Much has been published on the performance of germicidal UV across numerous applications and against different microbiological challenges.^10^ The test data available for the treatment of viral contaminants on surfaces continue to grow and have come under scrutiny during the SARS-CoV-2 pandemic. Some recent data indicate that, like other microorganisms, SARS-CoV-2 is highly susceptible to UVC irradiation.^1,10,11^

Even before this, earlier studies provided relevant information from other coronavirus data and showed a marked decline in infectious virus following UVC treatment.^12,13^

The fact that UV irradiation technologies are constantly evolving supports the use of standardized assessments for this type of equipment. Germicidal UV may, after all, offer a potentially valuable control measure for mitigating the spread of infectious viruses via surface contact. However, studies published to date differ widely in their approaches and have generated a range of efficacy data.^10^

This wide range of findings is likely due, in part, to the previous absence of a relevant national or international test standard, to ensure testing consistency. To help address this, a recently introduced British Standards Institution (BSI) test standard, BS 8628:2022 (hereafter referred to as BS 8628), was developed to provide a benchmark with which to assess UV irradiation device efficacy at room and cabinet scale.^2^

Here, although the testing approach has been aligned to the BSI method, the study was motivated by pandemic developments and related infection control questions. Therefore, rather than using the full BS 8628 suite of bacterial, fungal, and viral challenges, a well-established viral surrogate was used, bacteriophage Phi6. This enveloped, double-stranded RNA bacteriophage has been widely used as a surrogate for mammalian viruses, including the similarly enveloped SARS-CoV-2 virus.^14,15^

As with other bacteriophages, its low biohazardous nature allows a wider scope of viral challenge scenarios to be explored than would be possible with more hazardous viral agents, thus following the principle of hazard control by substitution. The use of surrogate bacteriophage is further supported, because these microorganisms typically offer an equally robust viral challenge when compared with mammalian viruses, and in some cases may exceed the UV resistance shown by some mammalian viruses.^13,16^

This work sought to assess the surface disinfection efficacy of two germicidal UVC emitting carousels at room scale. In addition, reusable powered air-purifying respirators (PAPR) were used as a test system to create a more challenging test scenario that included shadowing effects. This was intended to assess the UVC equipment using positional challenges that went beyond the basic test standard. A similar approach was taken when evaluating two UVC cabinets, where initial performance testing was aligned to the standard test method, followed by more challenging testing with PAPR equipment.

Most UV evaluation studies focus on the efficacy of the system, but safety is also an important aspect of UVC use as its uncontrolled exposure may harm the delicate membranes of the eyes and damage the skin.^3^ Of the systems tested here, the UVC carousels are not intended for use in the vicinity of people and must be used only in vacated areas. However, the tested cabinets are designed to shield the operator from UV exposure during use.

During the course of this work, the general usability and operational safety of the systems was monitored alongside germicidal performance. This included an assessment for potentially harmful ozone by-products, which have been linked to various types of high energy UV lamp systems.^17,18^ Although this is normally associated with UVC wavelengths of <240 nm, it was included here because some UVC lamps do not always emit energy solely at the optimal wavelength (254 nm).

## Methods

### UVC Devices Tested

Two UVC carousels and two UVC cabinets were tested. For the purposes of this report, we refer to the test equipment based on specification and design principles, rather than by brand name. The systems on test were as described in Table 1.

**Table 1.** UVC cabinet and carousel equipment tested

Device ID reference	Mode of operation	Additional comments	Supplier information—lamp output (W)	Measured UVC dose (average dose per minute based on 3 × 1 min readings)
1	Fully enclosed, bench top mounted UVC cabinet. Multi-directional UV delivery from lamps mounted on six sides	System has a small screen to show when the system is in operation. UV lamps cover all inner six surfaces. Internal dimensions: 348 mm *H* × 442 mm *D* × 477 mm W Weight: 80 kg	Each tube generates 6.9 UVC-W of UVC energy—Total UVC output = 276 UVC-W	2250 J/m^2^
2	Fully enclosed, bench top mounted UVC cabinet. Multi-directional UV delivery from lamps on top and bottom with reflective sides.	Small, UV opaque viewing window shows when system is in operation. Reflective material covering all six internal surfaces. Small carriers mounted on filter glass—Quartz based block; 180 × 140 mm. Internal dimensions: 250 mm *H* × 300 mm *D* × 500 mm W Weight: 17 kg	8 × 17 W UVC germicidal tubes (four above, four below). Total UVC output = 136 W.	2570 J/m^2^
3	Telescopic, upright 24 lamp UVC carousel system. Strictly for use in vacated rooms.	Tablet remote control system and motion sensors. External dimensions: 1490–2250 var. *H* × 670 W × 930 *D* (mm) Weight: 98 kg	Twenty-four × TUV 95 PL-L (SIGNIFY/PHILIPS) Each tube generates 27 W Of UVC energy—Total UVC output = 648 UVC-W	Range = 8340–10,620 J/m^2^
4	Upright UVC carousel with 10 irradiating lamps optionally controlled by Spectrome technology (UV spatial dosimeter control). Strictly for use in vacated rooms.	Approved card user security system and motion sensor. Remote-control touch screen system. External dimensions: 1880 *H* × 660 *W* × 610 *D* (mm) Weight: 72 kg	Ten low-pressure mercury lamps. Total power consumption, 1430 W at 230 V supply voltage. UVC-W output not available.	Range = 2842–5499 J/m^2^

UVC, ultraviolet C.

### Test Areas Used

All carousel work was undertaken in a fully enclosed 3 × 3 m blackout, non-reflective pop-up gazebo (model S30; TFH Gazebos, Ingatestone, United Kingdom). The tented structure provided sufficient interior space for the positioning of carousels and samples, allowing a safe distance between powered-up equipment and tent sides, while also aligning to positional requirements described in standard test BS 8628. The tent provided appropriate non-reflective interior surfaces for testing, protecting those outside of its perimeter from potentially harmful UVC radiation.

The two UVC cabinets were positioned on a standard worktop in a laboratory setting and used as required. Both were designed to be self-contained, with no possibility of UVC exposure for the operator once the interlocked door was closed and the machine activated.

### Surface Challenge Culture Preparation on Steel Carriers

The enveloped bacteriophage Phi6 (DSM 21518) was used for all challenge testing work. Fresh stocks of Phi6 were prepared by the propagation of existing frozen stocks in the recommended *Pseudomonas syringae* host culture (DSM-21482). The *P. syringae* culture was first prepared by adding 1 mL *P. syringae* suspension to 100 mL tryptone soya broth (Oxoid-Thermofisher) and incubating at 25°C in a shaking incubator set to 90 rpm for 18 h. A 100 μL aliquot of Phi6 was inoculated into the 18 h *P. syringae* culture and maintained overnight in the same conditions, to propagate the bacteriophage.

Following incubation, the Phi6 stock cultures were centrifuged at 3000 rpm (1690 *g*) for 10 min to remove cell debris. The supernatant was filtered consecutively through 0.45 and 0.2 μm filters (Millipore). The fresh Phi6 stock samples were serially diluted up to 10^−11^ to quantify the freshly cultured stock. One hundred microliters of each dilution was then mixed with *P. syringae* (18-h culture). The Phi6:*P.syringae* mix was added to 3 mL Tryptic Soy overlay, poured onto Tryptic Soy Agar plates, and incubated overnight at 25°C. Plaque-forming units were read from the plates the following day.

After determining the titer of Phi6 stock, the stock was further diluted in ¼ Ringer's solution supplemented with 0.3% bovine serum albumin (BSA; Sigma) at a dilution factor of 1/100, ready for pipette distribution on to sterile steel carriers. The solution also mimics “worse-case scenario” artificial sweat that contains interfering substances that could affect the efficacy of the treatment. This would be more in line with realistic contamination.

Just before each test, Phi6 suspensions were pipetted onto dry, steam sterilized, type 304 stainless steel carriers and used for all UVC testing. Because some tests required carriers to be positioned on/within small parts of PAPR equipment, the BS 8628 recommended carrier diameter of 40 mm could not be used as it was too large. Instead, the diameter was reduced to 22 mm and the volume of Phi6 suspension adjusted accordingly.

Based on the original recommended culture volumes in BS 8628, an amended volume of 12.5 μL of Phi6 bacteriophage was therefore applied, uniformly spread, and dried onto each steel carrier before these were dried at room temperature for 1.5 h in a biosafety cabinet.

### UV Cabinet Testing Procedure

For initial “baseline” testing, involving maximum UVC exposure in an approach aligned to BS 8628, carriers were placed horizontally and in triplicate within each of the UVC cabinets and exposed to UVC treatment. For System 2 the carriers were placed on a UV transmissible quartz plate due to the grated surface at the floor of the cabinet. Separate, otherwise identical samples were left untreated, in triplicate, as unexposed controls.

The UV treatment was repeated on 3 × triplicate sets of inoculated carriers. Both UV cabinet suppliers suggested an initial 1-min treatment as a starting point for assessing the performance of their equipment. However, improved log reductions of Phi6 were observed with longer treatments so treatment times were explored further (data not shown).

A 4-min treatment was the maximum programmable treatment delivery period possible with System 1 in a single treatment cycle, although treatments of up to 5 min were assessed (4 min continuous plus an additional minute applied immediately after). It was determined that the benefits of >4 min of treatment were minimal and 4-min treatment was therefore settled on as the default treatment period for all tests using System 1.

System 2 had a maximum programmable treatment time of one minute and therefore had to be re-run multiple times to achieve a total treatment UVC dose to match that of System 1; based on measured dose, this equated to three × 1-min treatment deliveries for each complete treatment using System 2.

As a variation from BS 8628, additional assessments were then undertaken using re-usable PAPR ensembles. These tests were designed to incorporate a combination of both shadowed and more exposed carrier locations using these compact equipment items, to see how effectively the viral contaminants could be treated. For each test, the same type of steel carriers was located in sets of three at multiple positions using adhesive tape, as reported for previous decontamination work,^19^ around the equipment, with three different types of PAPR employed for this purpose.

The PAPR items used were all widely available models sold internationally: PAPR A, PAPR B, and PAPR C with corresponding pumps appropriate to their fittings (Table 2). All tested equipment had accompanying corrugated hose attachments from hood/helmet to pump. Common carrier locations were used across all three PAPR equipment types, and given a letter code for reporting purposes, as follows:

**Table 2.** Powered air-purifying respirator equipment with descriptions of equipment type

PAPR equipment identifier	Head piece description	Pump description
A	Hooded Headpiece, no hard hat inside. Visor at the front, pump connection at the back. Corrugated hose attached.^a^	Standard pump with removable filter—inlet at the side.
B	Hooded Headpiece with hard hat inside.	Standard pump with removable filter—inlet at the side. Corrugated hose attached.
C	Hard hat with visor, material around bottom to produce a seal.	Standard pump with removable filter—inlet at the side. Corrugated hose attached.

^a^ Hood A had the hose permanently attached; therefore, the corrugated hose was tested with the hood instead of the pump.

PAPR, powered air-purifying respirator.

- inside the headpiece visor; V
- top front face of pump; O
- inside the head band; M
- on the inner side of longer part of hood; K
- around the inner part of clean air outlet of pump; I
- within the concertina structure of the flexible clean airflow hose; H
- at the deepest point on the inner side of hood; C

Each test was repeated three times and due to the collective size of the PAPR components, hoods/headpieces, hoses, and pumps were processed separately within each cabinet, with each receiving the same UVC treatment within a test period of 15 min.

### UV Carousel Testing Procedure

Each carousel was tested in the large, blacked out non-reflective tent, providing “baseline” conditions that were based on uninterrupted UVC exposure for Phi6 samples, that is, no imposed shadowing effects, using the exact approach described in BS 8628. Briefly, the challenge carriers were positioned horizontally on a flat, non-reflective plinth at 1 m height and at a distance of 2 m from the carousel UVC source face.

To further evaluate the “line of sight” related performance of each carousel system, additional carrier positions were introduced (not required for BS 8628), whereby triplicate samples were placed vertically on the same plinth, with the biological challenge material facing the carousel. This latter approach was intended to determine the extent of any treatment variation between the horizontally and vertically positioned carriers, based on the rationale that any room contains both types of surface orientation.

Each carousel was run for 25 min duration per treatment, as agreed with the suppliers, and any reduction in microorganism levels on carrier surfaces after this period was determined as described next.

The original plan for carousel testing was to undertake the work exactly as described in BS8628, that is, without any PAPR equipment props. However, after discussions with one of the carousel suppliers, it was decided to include a wider assessment approach, again using re-usable PAPR equipment, though in this instance using only one ensemble type (PAPR B).

The PAPR carrier location positions were as described for the cabinet scale testing but for carousel work, PAPR equipment was suspended using fine cords, exactly 1 m from the front face of each carousel. The hose/headpiece were hung as one item and the heavier pumps components suspended separately, and adjacent to other ensemble items.

The suspended PAPR items were orientated so that the sample carriers were in line of sight of the UVC emission, with the convoluted surfaces of the PAPR still providing inherent shadowing effects on the treated surfaces, compared with the more openly exposed plinth tests.

### Sample Processing and Quantification—Cabinet and Carousel Carrier Challenges

Following each treatment process, each triplicate set of UV-treated carriers and untreated controls were retrieved and immediately immersed in 5 mL of sterile phosphate-buffered saline (PBS), with each sample tube containing two sterile glass beads. Each tube was vortexed for 30 s and left at room temperature for 30 min to allow phage deposits to dissociate from the carrier surface.

After a further brief mixing, 100 μL of the sample was serially diluted from Neat (0) to 10^−5^ dilution in sterile PBS. One hundred microliters of each dilution fraction was mixed with 300 μL of 18 h cultured *P. syringae*. The inoculated *P. syringae* mixture was added to 3 mL Tryptic Soy Overlay agar, poured onto Tryptic Soy Agar plates, and incubated overnight at 25°C. Any formed bacteriophage plaques were counted the following day, typically 16 h after plating out.

### Ozone By-Product Measurement Close to UVC Devices

Ozone by-product generation close to carousel UV lamps was tested for using a 2B Technologies Model 205 Dual Beam Ozone Monitor, accurate to within 1 part per billion (ppb). The pre-calibrated monitor was located outside of the test structure and appropriate diameter Teflon™ tubing was run in to the test area via an access port, with the tip of the tubing placed within 10 cm of the carousel lamps. Ozone levels were logged constantly, for the duration of each test. Cabinets were less easy to assess, because the door seals on the cabinets were found to constrict any tubing inserted therein.

To circumvent this, the ozone measurement equipment was set up, running, alongside each cabinet and used to immediately measure the air at the face of the cabinet door when opened after each period of treatment. The rationale for this was that any ozone emission would accumulate within the cabinet space during use and be detectable if present. The mode of measurement here, therefore, mimicked potential operator exposure to ozone by-products as it might occur as they removed treated items from the cabinet.

### UVC Irradiance and Dosage Measurement

Irradiance and dose measurements were taken during carousel tests using a newly calibrated digital radiometer (RMD special version, UVC, Opsytec, Dr Gröbel), Mannheim, Germany. This instrument offered instant, real-time measurement but did not log data. It proved useful for carousel work in the blackout tent, but its sensor connector cable made it less suitable for use within the UVC cabinets (the cable impeded cabinet door closure).

The UVC dosage measurements within UVC cabinets were more easily measured using a newly calibrated UV dose meter (UV Light Technologies, Birmingham, United Kingdom). This compact instrument showed accumulated dosage data, which could also be read via the small viewing screens/aperture available on the UV cabinets.

### Data Analysis

For UVC cabinet testing, any reduction in microorganism levels after the described surface treatment was measured using log reduction analysis, which was calculated as log_10_(unexposed surface concentration) minus log_10_(exposed surface concentration). The unexposed controls were prepared under identical conditions to the test carriers and stored under equivalent conditions for the duration of the test but not exposed to UV. The log reduction data were summarized by showing measures of central location (i.e., mean and median) and spread (i.e., standard deviation, minimum, maximum, and inter-quartile range) of the distribution, in both tabular and graphical formats.

For UVC carousels, any reduction in microorganism levels was assessed after 25 min of surface treatment and again measured using log reduction analysis, which was calculated as log_10_(unexposed surface concentration) minus log_10_(exposed surface concentration). The unexposed controls were prepared under identical conditions to the test carriers and stored under equivalent conditions for the duration of the test but not exposed to UV.

The log reduction data were again summarized by showing measures of central location (i.e., mean and median) and spread (i.e., standard deviation, minimum, maximum, and inter-quartile range) of the distribution, in both tabular and graphical formats.

Log reduction data for different groups of cabinets, carousels, PAPR systems, and PAPR positions were compared in turn. Various statistical tests are available to compare groups of data, and parametric methods (e.g., linear regression models/analysis of variance) were used where possible as these are more informative. However, certain criteria must be fulfilled (e.g., normally distributed data, the variances of the group populations are equal) in order for these to provide reliable results. The relevant conditions were tested in each case.

For instance, the Levene test was used to determine equality of variances, and the Shapiro–Wilk test and plots of standardized residuals were used to determine whether data were normally distributed. Where the criteria were not sufficiently fulfilled, non-parametric tests (e.g., Wilcoxon Rank Sum, Kruskal–Wallis Rank Sum, Dunn's Test) were used instead.

In some instances (e.g., where there was a systematic change in the spread of residuals over the range of measured values), techniques such as linear regression with robust standard errors could be used to obtain more informative results, especially when determining the extent of interaction between the input variables. When expressing differences in log reduction, a best estimate of the difference, along with a confidence interval (95% CI), were provided whenever possible.

All analysis was performed using R statistical software.^20^

## Results

### Dosage Values Measured for the UV Systems

Measured UVC dosage values are given for cabinets and carousels in Table 1, alongside system specifications.

### Ozone Measurements

No detectable ozone by-products were measured in the vicinity of the UV test equipment at any time during their use.

### Cabinet Results

**Figure 1.**
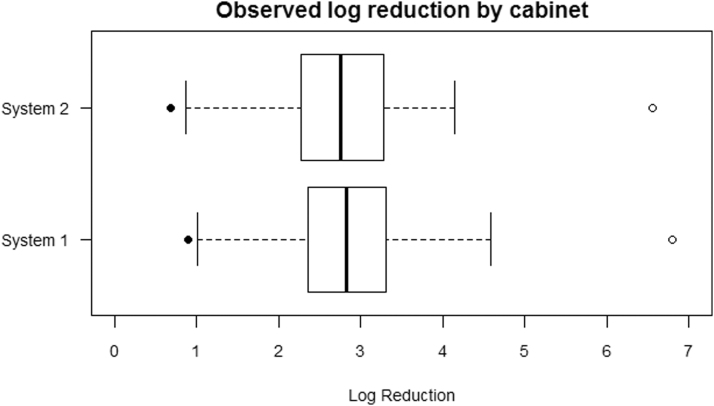
Observed log reduction and mean log (unexposed concentration) values by cabinet (no PAPR present). The log reduction values are represented by the box and whiskers, with outlying points shown as solid circles. The hollow circles represent the mean log (unexposed concentration) values. PAPR, powered air-purifying respirator.

Log reduction values (after 4 mins treatment with System 1; 3 min with System 2) for each UV cabinet across the three experimental runs are shown graphically in Figure 1 in the form of a box plot, where log reduction values are represented by the box and whiskers with outlying points, which differ substantially from the rest of the distribution, shown as solid circles.

The log_10_(unexposed concentration) values are represented by hollow circles. The unexposed concentration represents an upper “ceiling” for the maximum achievable log reduction. The log reduction figures for System 1 were slightly higher than those for System 2, and this may be partly due to the differing unexposed concentrations. Numerical data for log reduction values are summarized in Table 3.

**Table 3.** Summary of log reduction values and the mean log (unexposed concentration) values for cabinet and carousel systems

System	No. of observations^a^	Log reduction summary						Mean log (unexposed concentration)^b^
		Mean	Standard deviation	Min.	Median	Inter quartile range	Max.	
Cabinet 1	63	2.80	0.77	0.90	2.82	0.96	4.58	6.79
Cabinet 2	63	2.66	0.80	0.69	2.76	1.02	4.15	6.56
Carousel 3	27	2.87	0.67	1.02	2.80	0.58	4.59	6.33
Carousel 4	27	2.37	0.44	1.40	2.30	0.48	3.45	6.29

^a^ Each observation is derived from challenge carriers seeded with the Phi6 bacteriophage challenge organism.

^b^ log (unexposed concentration) represents a “ceiling” for the maximum log reduction achievable.

There was little difference between the two cabinets based on data presented, in terms of both the central location and the spread of the data. A Wilcoxon Rank Sum test was applied to compare these groups. This test found no statistically significant difference (*p* = 0.50) in the median log reductions achieved by the two cabinet systems. This was despite the systems being supplied by different companies and having slightly different technical specifications.

### Combination of Cabinet and PAPR System

**Figure 2.**
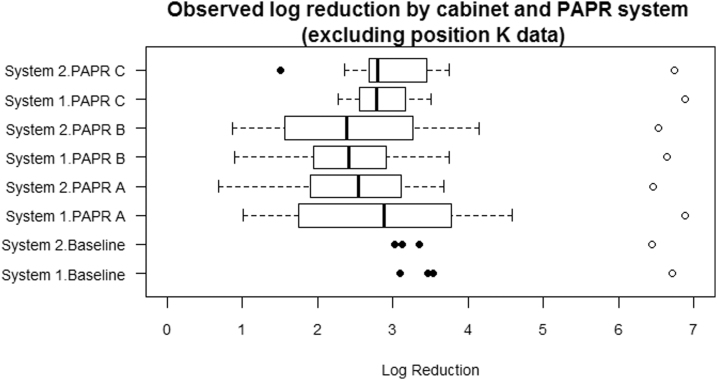
Observed log reduction and log (unexposed concentration) values by cabinet and PAPR system. The log reduction values are represented by the box and whiskers, with any outlying points shown as solid circles. The hollow circles represent the log (unexposed concentration) values. The “baseline” data represent tests where no PAPR equipment was placed in the cabinet.

Figure 2 shows the log_10_ (unexposed concentration) and log reduction for each combination of cabinet and PAPR system. The additional inclusion of “baseline” data within the figure represents tests where no PAPR equipment was placed in the cabinet. There are indications of some differences in log reduction values for the various combinations of cabinet and PAPR systems.

The analysis for cabinets alone showed that the difference between the two systems was not statistically significant. The analysis for PAPR systems alone showed that the baseline was associated with the highest log reductions followed by PAPR C, A, and finally B. When the baseline data were omitted, and the PAPR systems were considered in isolation, the Kruskal–Wallis rank sum test gave a borderline significant result (*p* = 0.071) for the difference in log reduction between the groups.

Comparing the groups in pairs, Dunn's Test (with a Bonferroni–Holm multiplicity correction) showed that the difference in log reduction for PAPR B/PAPR C was borderline significant (*p* = 0.066), whereas for both of the other pairs it was *p* = 0.35. These results are reflected in Figure 2.

When a linear regression model with robust standard errors featuring both cabinet and PAPR system was fitted, this confirmed that the two cabinet systems are not significantly different (*p* = 0.66) and that all PAPR systems produced significantly different log reductions than baseline: PAPR A (*p* = 0.001), PAPR B (*p* < 0.001), and PAPR C (*p* = 0.010).

On average, the log reduction was 0.65 lower (95% CI 0.26–1.03) for PAPR A than baseline, 0.83 lower (95% CI 0.47–1.18) for PAPR B, and 0.43 lower (95% CI 0.11–0.75) for PAPR C than baseline.

There is no appreciable evidence of interaction, where the log reduction for a combination of cabinet and PAPR system is substantially different from that expected based on the effects of cabinet and PAPR system considered separately. When an interaction term was incorporated, it was found that none of the interaction effects were statistically significant (lowest *p* = 0.33). These findings are consistent with the initial impressions based on Figure 2.

### Combination of PAPR System and PAPR Position

The log_10_ (unexposed concentration) and log reduction for each combination of cabinet and PAPR position are not shown here due to the large number of possible combinations of these two variables.

The PAPR system and position data are correlated in that the position variable can only take a value of C, H, I, M, O, or V if the PAPR system is not “baseline.” It was not possible to accurately determine the effects of the different PAPR systems and positions if these two variables are included in the same model whereas there is correlation between them. However, by excluding the baseline data from this particular analysis, it was possible to circumvent the issue of correlation.

The analysis for PAPR systems alone showed that PAPR C was associated with the highest log reductions followed by systems A and finally B. The analysis for PAPR positions alone showed that positions C (inner hood), M (headband), and V (visor) were found to be different from some of the other positions.

When a linear regression model featuring both PAPR system and position was fitted, this showed that the log reduction for PAPR C was significantly different (*p* = 0.038) from PAPR B, whereas PAPR A was not (*p* = 0.41). On average, the log reduction for PAPR C was 0.29 (95% CI 0.019–0.56) higher than for PAPR B.

Also, PAPR positions M and V were significantly different (*p* = 0.001) than position C, whereas all other positions were highly significantly different (*p* < 0.001). On average, compared with position C, the log reduction for position H (hose) was 1.36 (95% CI 1.00–1.72) higher, for position I (inner rim, clean air outlet) 1.23 (95% CI 0.86–1.59) higher, for position K (inner hood) 1.48 (95% CI 1.07–1.89) higher, for position M (headband) 0.61 (95% CI 0.25–0.97) higher, for position O (face of pump) 1.70 (95% CI 1.34–2.06) higher, and for position V (visor) 0.62 (95% CI 0.26–0.98) higher.

There was some evidence of interaction, where the log reduction for a combination of PAPR system and position was substantially different from that expected based on the effects of PAPR system and position considered separately. When an interaction term was incorporated, the following interaction effects were statistically significant: PAPR C position H (*p* < 0.001), PAPR C position O (*p* = 0.004), and PAPR C position V (*p* = 0.008).

PAPR A position V was of borderline statistical significance (*p* = 0.054). On average, the log reduction for PAPR C position H is 1.34 (95% CI 0.57–2.11) lower than expected, for PAPR C position O is 1.19 (95% CI 0.41–1.98) lower than expected, and for PAPR C position V is 1.07 (95% CI 0.30–1.84) lower than expected.

### Combination of Cabinet, PAPR System, and PAPR Position

As in the previous section, the baseline data have been excluded from this particular analysis, to circumvent the issue of correlation between PAPR system and position. The optimal linear regression model featuring a combination of these three variables was found to include the following: cabinet, system, position, the interaction of cabinet and system, and the interaction of system and position.

When fitted, this model gave very similar results to the model fitted for the combination of PAPR system, PAPR position, and their interaction. The only statistically significant variables were those featuring the PAPR system and PAPR position. Put simply, this confirms that the most important variables for predicting log reduction are the type of PAPR system, the PAPR part (location) being treated, and the interaction of these two variables. Cabinet type was less important based on this reported comparison test.

## Carousel Results

**Figure 3.**
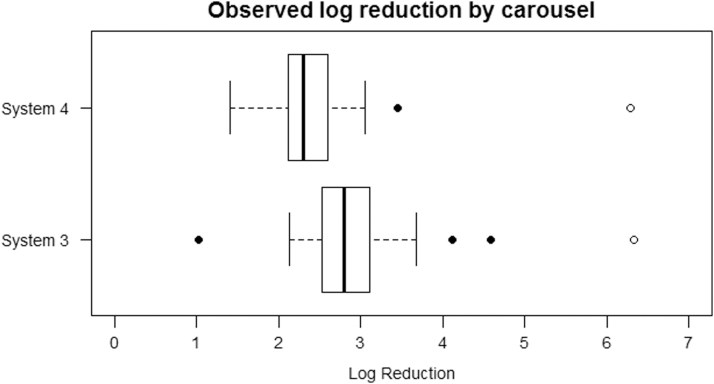
Observed overall log reduction and mean log (unexposed concentration) values by carousel type. The log reduction values are represented by the box and whiskers, with outlying points shown as solid circles. The hollow circles represent the mean log (unexposed concentration) values.

Log reduction values (obtained after 25 mins exposure) for each UV carousel across the three experimental test runs (including baseline runs) are shown graphically in Figure 3 in the form of a box plot, where log reduction values are represented by the box and whiskers with outlying points shown as solid circles. The log_10_(unexposed concentration) values are represented by hollow circles. Numerical data for log reduction values are summarized in Table 3.

The unexposed concentration represents an upper “ceiling” for the maximum achievable log reduction. The mean log (unexposed concentration) figures for System 3 and System 4 are similar. However, the log reduction figures for System 3 are typically higher than those for System 4, so the unexposed concentration figures cannot account for this difference. Also, the spread of the System 3 data is wider.

To determine whether there is a statistically significant difference in the mean values of log reduction of the two groups, the Wilcoxon Rank Sum test was applied. This test identified a significant difference (*p* < 0.001) in the median log reductions achieved by the two carousel systems. On average, the log reduction for System 3 is 0.48 (95% CI 0.16–0.81) higher than for System 4.

### Combination of Carousel and PAPR System

**Figure 4.**
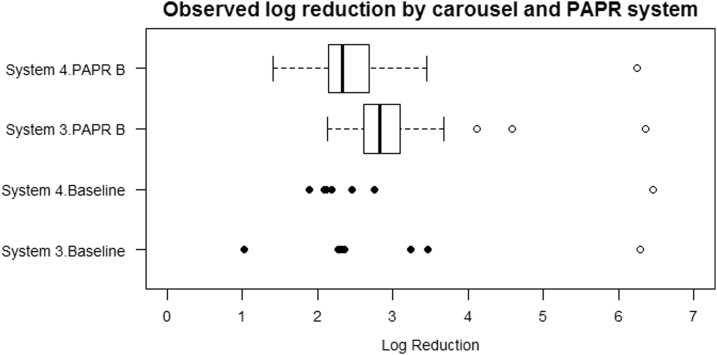
Observed log reduction and log (unexposed concentration) values by carousel and PAPR system. The log reduction values are represented by the box and whiskers, with outlying points shown as solid circles. The hollow circles represent the log (unexposed concentration) values. The “baseline” data represent tests where no PAPR equipment was used.

Figure 4 shows the log_10_(unexposed concentration) and log reduction for each combination of carousel and PAPR system. The “baseline” data represent tests where no PAPR equipment was used.

There is an indication of some differences in the distributions of log reduction values for the various combinations of carousel and PAPR systems. The analysis for carousels alone showed that System 3 produced significantly higher log reductions than System 4, whereas the analysis for PAPR systems alone showed that PAPR B was associated with higher log reductions than Baseline, and the difference was of borderline statistical significance (*p* = 0.070). Figure 4 appears to reflect these same trends.

When a linear regression model with robust standard errors featuring both carousel and PAPR system was fitted, this showed that System 3 produced significantly different log reductions than System 4 (*p* = 0.013) and that PAPR B was associated with higher log reductions than Baseline but the difference is not statistically significant (*p* = 0.12). On average, the log reduction for System 3 is 0.44 (95% CI 0.10–0.79) higher than for System 4.

There is no appreciable evidence of interaction, where the log reduction for a combination of carousel and PAPR system is substantially different from that expected based on the effects of carousel and PAPR system considered separately. When an interaction term was incorporated, it was found that any interaction effects were not statistically significant (for System 3: PAPR B, *p* = 0.85), and this is consistent with initial impressions based on Figure 4.

### Combination of PAPR System and PAPR Position

The PAPR system and position data are correlated in that the position variable can only take a value of C, H, I, K, M, O, or V if the PAPR system is not “baseline.” It is not possible to accurately determine the effects of the different PAPR systems and positions if these two variables are included in the same model whereas there is correlation between them.

For the cabinet data, it was possible to circumvent the issue of correlation by excluding the baseline data. However, if the same were to be done for the carousel data, there would only be one PAPR system left in the data, meaning that it would be impossible to determine the effect of PAPR system on log reduction or the effect of interaction between PAPR system and position. Therefore, no analyses were possible for the carousel data where both PAPR system and position were included. Instead, PAPR position alone was analyzed next.

### PAPR Position

**Figure 5.**
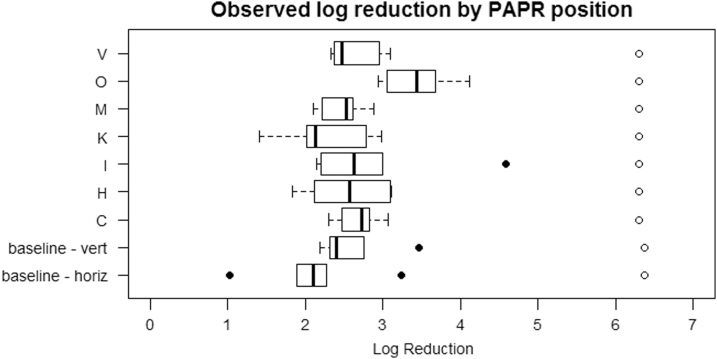
Observed log reduction and log (unexposed concentration) values by PAPR position. The log reduction values are represented by the box and whiskers, with outlying points shown as solid circles. The hollow circles represent the log (unexposed concentration) values. The “baseline” data represent tests where no PAPR equipment was used. The position codes are again defined as follows: V = Visor; O = Top front of pump; M = Head band; K = Inner side of longer part of hood; I = Inner part of outlet; H = Hose; C = Top of inner side of hood.

Figure 5 shows the log_10_(unexposed concentration) and log reduction for each PAPR position. The “baseline” data represent a series of tests where no PAPR equipment was used.

Figure 5 indicates some differences in the distributions of log reduction values for the various PAPR positions, and the Kruskal–Wallis test indicated a significant difference between some of the positions (*p* = 0.022). Comparing the groups in pairs, Dunn's Test (with a Bonferroni–Holm multiplicity correction) showed that the difference in log reduction for position O/position K was significant (*p* = 0.029) as was position O/baseline-horizontal (*p* = 0.007), whereas all other pairs were not significantly different (minimum *p* = 0.32).

Of the two baseline categories that were assessed, vertically or horizontally mounted carriers, the vertically mounted carriers gave a noticeably higher overall level of log reduction, as might be expected from their more direct line of UV exposure.

## Discussion

This work was conducted to apply standardized testing procedures to determine the efficacy of UV treatment devices on bacteriophage surrogates, with or without the presence of “real world” respiratory protective equipment. The initial baseline assessments were thus extended beyond the scope of standardized system evaluation, to include more complex testing using PAPR devices that would generate challenging surfaces to disinfect using UVC.

The focus of the work was on the efficacy of reducing bacteriophage Phi6, and this microorganism was selected for its similar size and structure to SARs-CoV-2, with its minimal hazard to humans making it easier to handle for experimental purposes.

Each carrier used for this testing was seeded with a “worst case scenario” artificial sweat that contained not only the Phi6 but also a mixture of BSA, an interfering substance that could conceivably interfere with the efficacy of UV treatment. This was to more closely mimic general day-to-day contaminants that would be transferred onto equipment and to reflect the fact that viral transfer alone is unlikely to ever happen in the real world.

With the real-world application in mind, it is also important to note that for most disinfection treatments, including UVC, it is recommended to first physically clean any surface, before the disinfection step. Indeed, the application of UVC would be most sensibly and effectively applied as an enhanced hygiene step, once physical cleaning and wet disinfection of equipment have already been completed.

### Cabinets

When assessing the efficacy of the UV Cabinets, the testing first considered standard “baseline” tests, with only seeded carriers inside the cabinet. Both cabinets demonstrated a measurable and similar log reduction after 3 to 4 min treatments (max log reduction ∼4.58).

British standard BS 8628 stipulates that, for UV cabinet and carousel devices, virucidal activity against bacteriophages should give a 4-log or greater reduction on test carriers compared with control carriers that have not been exposed to the process for the strains described. Some of the results here, therefore, fell within that requirement.

When comparing the two individual cabinets, the log reduction figures for System 1 were slightly higher than those for System 2. This may be partly due to the differing unexposed concentrations, that is, the starting challenge for each series of baseline tests. However, there are other influences that can affect Phi6 recovery, such as relative humidity and temperature.

It is likely that these variables account for some differences in log reduction values for both UVC cabinets and UVC carousels, despite every effort to ensure that repeat tests were undertaken in near identical fashion. Although every effort was made to keep test conditions consistent between tests, there is always inherent variation that cannot be accounted for.

Both cabinets offered a similar delivery of UV dose, although they differed in the exact mechanism of delivery. For example, System 1 contained UV bulbs on all six internal walls of the cabinet, whereas System 2 contained UV bulbs at the top and bottom of the cabinet and used reflective surfaces to facilitate uniform delivery.

Statistically, there appeared to be little difference between the two cabinets in terms of both the central location and the spread of the data. Both systems, therefore, demonstrated similar performance despite their slightly different technical specification.

When considering the added level of protective element (BSA) of the artificial mixture used on the carriers for this testing, the maximum observed 4.58 log bacteriophage reduction demonstrated a measurable level of efficacy. The method of seeding carriers may add further to the protection of bacteriophage on carriers, for example, due to a layering effect where high titers are used, which may confer physical protection for underlying surface deposits of phi6.

Based on the findings here, UVC cabinet treatments could help to reduce viable virus levels and hence the transmission potential of viruses on the surfaces of reused items of equipment, such as PAPR, particularly when used in combination with physical surface cleaning and disinfection.

### Carousels

The carousel tests were aligned with the BSI standard, using a non-reflective black test facility to limit reflective/absorbing surface that could otherwise interfere with the UV dose delivered. The highest log kill for the carousels was between 3.45 and 4.59 log. Again, some of the results obtained here therefore fell within the BS 8628 requirement.

Generally, the log reduction figures for System 3 were higher than those for System 4 and there was a statistically significant difference in the reductions between the two. From the UVC dose data, it is evident that System 3 delivered an almost twofold higher dose of UVC compared with System 4. Therefore, more UV irradiance was delivered to carrier surfaces, from the UV source.

This is the most likely explanation for the difference in performance between the two systems and may have been further enhanced by the ability of System 3 to vertically self-adjust and extend its lamp height during use, with the supplier claiming that its 2.25 m maximum height could benefit UV dose delivery to the targeted surfaces.

Since the carousels were from different suppliers, there were other differences in their mechanisms of action. For example, System 3 was able to scan and map the room to determine its size and to then calculate optimal lamp height and treatment time based on those self-generated data, although that mechanism could be manually overridden.

System 4 used probes located around the room to ensure the delivered UV reached each location and would adjust the treatment time until all probes received the required dose. Again, this could be overridden if a manually set treatment period was preferred. Before testing, each carousel was set to the room specifications using its respective automated UV delivery settings and UVC treatment duration was provided for each using this approach.

Initial pilot testing, using a small number of test carriers, suggested that this was not generating the expected reductions in Phi6 target organisms (data not shown). To avoid inconsistency, a treatment duration of 25 min for each test was used and this was pre-agreed with both carousel suppliers. This exceeded the treatment period suggested by the software of both systems and was felt to be more realistic in terms of the efficacy levels sought from the testing.

### PAPR System Decontamination

Different types of PAPR system were used here to provide a challenging test scenario and thus generate a more complex model whereby it would be possible to assess potential real-world applications of the UVC devices.

For the two cabinets, the baseline values for log reduction, based on carriers being fully exposed to UVC with no shadowing effects, were higher than those for the PAPR systems. The introduction of PAPR equipment provided a more complex treatment challenge for the UVC systems, including variable carrier orientation and different levels of shadowing as a result of the various recesses and material surfaces associated with the PAPR equipment.

During cabinet testing, small differences were observed between the level of log reduction for different types of PAPR systems and slightly differing unexposed concentration values may have contributed to this effect, as could slight differences in the distance of seeded carriers from the UVC source when placed on/within various parts of the PAPR equipment.

The statistical analysis demonstrated that overall, all PAPR systems produced a significantly lower log reduction than baseline. On comparing the PAPR systems pairwise, the difference between PAPR B and PAPR C was of borderline significance, with PAPR C producing higher log reductions than PAPR B, and PAPR A between the other two. This suggests that the intricacies of equipment design may play some part in how effectively such equipment can be treated with UV, since every effort was made to ensure that carrier locations were consistent between PAPR systems.

When assessing the performance for each combination of cabinet and PAPR system, there was no significant difference from that expected, based on the effects of the cabinet and PAPR system considered separately.

For the carousels, only the PAPR B ensemble was selected for testing and compared against the baseline (no PAPR in situ). After discussion with carousel suppliers and consideration of the available test space, the PAPR ensemble was suspended 1 m away from the UVC lamps for each test.

The log reduction for samples located on and within PAPR B was subsequently found in many cases to be higher than the baseline log reductions, though overall the difference was not statistically significant. A contributing factor was almost certainly that the baseline measurements for the carousels were taken in accordance with the BSI standard, that is, 2 m away from the UV source. The baseline carriers were, therefore, positioned more distant from the UVC source than PAPR-mounted samples and the latter would have received a higher UV dose.

The fact that the PAPR inoculum would have experienced a greater degree of shadowing did not prevent the overall log reduction of bacteriophage on the PAPR being greater than Baseline values, but in particular when considering the horizontally mounted Baseline carriers.

When assessing the performance for each combination of carousel and the PAPR B system, there was no significant difference from that expected based on the effects of the carousel and PAPR system considered separately.

### The Influence of PAPR Position

When considering the individual sample points on the PAPR systems, the statistical analysis showed a significant difference in the log reduction between sample locations. This was to be expected due to shadowing effects, such as the recesses of the equipment that would prevent a clear line of UVC delivery to the individual sample position.

When considering the results of the individual sample points, position O had the highest level of log reduction as would be expected as this corresponded to the smooth pump surface and was completely exposed to UVC. The log reduction for position O was also slightly higher than that of the baseline. This is likely due to position O being more prominent in the cabinet, closer to the UV source and therefore experiencing greater bacteriophage Phi6 reduction as a result of a marginally increased UVC dose (compared with baseline).

Positions C, M, and V were inside the PAPR headpiece and therefore subject to much greater interference from shadowing effects. Incrementally, the results correspond to the distances these positions were situated from the outside of the headpiece. Position V was just inside the visor, and though shadowed it would have been closer and more exposed to the UVC emission source.

Position M was just inside the headband, again slightly shadowed but still in close proximity of the UVC source. Position C had the highest degree of shadowing, further inside the headpiece and the furthest distance from the UV source. As such, the log reduction for position C was significantly different from that for all other positions.

There was some evidence of interaction, where the log reduction for a combination of PAPR system and position is substantially different from that expected based on the effects of PAPR system and position considered separately. In particular, the log reduction for PAPR C positions H, O, and V were significantly lower than expected.

The results of the PAPR positions for the carousels showed some similarity to those for the cabinets. Statistically, the PAPR data from the carousels showed more consistency between tests, indicating that decontaminating PAPR with a carousel would likely be more consistently effective than using a cabinet system. The most likely explanation for this is that the PAPR system used for carousel testing was exposed to a higher dose of UV for a prolonged period of time, compared with cabinet treatments.

The log reduction for position C is substantially higher for the carousels than for the cabinets, whereas the log reductions for position K and baseline are notably lower for the carousels than for the cabinets. Comparing the positions pairwise, the only significant differences found were between position O and baseline (horizontal), and between position O and position K.

These observations are most likely a combination of the fact that, for baseline testing, the standard test procedures specified for different UV equipment differed, as demanded by their design, that is, carriers were located more distantly from the carousel UV source, compared with cabinets and for carousels the UVC emission was hitting the carriers from one direction whereas treatment was multi-directional for cabinets.

It is, therefore, not possible to compare with like between carousel and cabinet tests, despite them all delivering UVC. Any differences in PAPR equipment treatment outcomes would almost certainly be the result of different positional procedures, necessitated by the fact that for cabinets, PAPR was simply placed within the cabinets, a minimal distance from UVC lamps. By contrast, for carousels PAPR items had to be suspended a safe distance from carousels, to effect treatment.

### Device Safety in Use

Germicidal UVC is hazardous and capable of damaging the eyes and skin if they are exposed, even for short periods. As such, instruments delivering UVC should be designed in such a way as to make them inherently safe to use. All of the systems tested here had design features that made accidental UV exposure unlikely. The UVC carousels must only be used to treat unoccupied rooms and both devices were fitted with motion sensors, which automatically switched the device off, for example, if a room door opens or if someone approaches the device.

The carousels were controllable from outside the treated area using remote device software, removing the need to be close to the machines as they were activated. In support of these features, both devices are supplied with comprehensive user training at the point of end-user delivery, to further reduce the likelihood of misuse and UVC exposure.

Both UVC cabinets comprised sealed units with front access doors, allowing no exposure to UV during the treatment period. Each unit operated with an interlock system, allowing cabinet door opening only once UV irradiation delivery had ceased. In addition, System 1 had audio prompts to confirm when UV treatment had started and finished. Comprehensive user training was again available from both suppliers, although these smaller units were of simpler designs than the larger carousels, with key operation skills learned in minutes.

## Conclusions

There is limited, consistent research regarding the ability of germicidal UVC technologies to treat SARS-CoV-2, or an appropriate surrogate, as part of a side-by-side comparison study. This work reports such a study for two UVC cabinets and two UVC carousel devices, with equipment either bench standing (cabinets) or floor mobile on wheels (carousels). Under standardized test conditions, all test devices provided measurable baseline reductions in bacteriophage Phi6 concentration when optimal exposure test conditions were applied and when shadowing effects were minimal or absent.

The devices were further evaluated with inoculated carriers positioned at various locations on or within PAPR ensembles, a widely used type of reusable, respiratory protective equipment. Germicidal performance varied under these latter test conditions, but measurable reductions in bacteriophage were detected for all test devices, even in more recessed areas of the PAPR ensembles.

Overall, when using a high level of Phi6 challenge, UVC cabinets and carousels demonstrated levels of bacteriophage reduction that could potentially augment routine hygiene measures used for PAPR, such as wet physical cleaning. This suggests that, in real-world scenarios, germicidal UVC devices could help to mitigate viral pathogen spread via reused equipment and may offer benefits for reducing the surface transmission of viruses such as SARS-CoV-2. The relatively modest cost of UVC cabinet systems and short treatment times makes them particularly accessible within the context of health care and industrial use, where smaller equipment items may require surface disinfection following routine cleaning.

Further work would be required to explore the full potential of using cabinet based UVC devices for treating other small items of equipment that might otherwise act as transmitters of infectious disease. In addition, our study of PAPR treatment using carousels was limited in scale, using only one type of PAPR ensemble, and would require higher levels of sample testing to underpin these initial data.

## Authors' Contributions

A.B.: conceptualization, methodology, validation, investigation, and writing (original draft, review, and editing). J.B.: methodology, validation, investigation, and writing (original draft, review, and editing). I.R.: validation, investigation. C. Bailey: validation, investigation. C. Beynon: validation, investigation. S.S.: investigation. N.B.: formal analysis.

## Disclaimer

Its contents, including any opinions and/or conclusions expressed, are those of the authors alone and do not necessarily reflect Government or HSE Policy. All UVC systems were loaned cost-free by the supplying companies. © Crown Copyright 2023.
